# Image-guided neural activity manipulation with a paramagnetic drug

**DOI:** 10.1038/s41467-019-13933-5

**Published:** 2020-01-09

**Authors:** Sarah Bricault, Ali Barandov, Peter Harvey, Elizabeth DeTienne, Aviad Hai, Alan Jasanoff

**Affiliations:** 1 Department of Biology, Massachusetts Institute of Technology, 77 Massachusetts Ave. Rm. 16-561, Cambridge, MA 02139 USA; 2 Department of Biological Engineering, Massachusetts Institute of Technology, 77 Massachusetts Ave. Rm. 16-561, Cambridge, MA 02139 USA; 3 Department of Electrical Engineering & Computer Science, Massachusetts Institute of Technology, 77 Massachusetts Ave. Rm. 16-561, Cambridge, MA 02139 USA; 4 Department of Brain & Cognitive Sciences, Massachusetts Institute of Technology, 77 Massachusetts Ave. Rm. 16-561, Cambridge, MA 02139 USA; 5 Department of Nuclear Science & Engineering, Massachusetts Institute of Technology, 77 Massachusetts Ave. Rm. 16-561, Cambridge, MA 02139 USA

**Keywords:** Magnetic resonance imaging, Neural circuits, Chemical tools

## Abstract

Targeted manipulations of neural activity are essential approaches in neuroscience and neurology, but monitoring such procedures in the living brain remains a significant challenge. Here we introduce a paramagnetic analog of the drug muscimol that enables targeted neural inactivation to be performed with feedback from magnetic resonance imaging. We validate pharmacological properties of the compound in vitro, and show that its distribution in vivo reliably predicts perturbations to brain activity.

## Abstract (web summary)

Targeted manipulations of neural activity in the living brain remain a significant challenge. In this study the authors introduce a paramagnetic analog of the drug muscimol that enables targeted neural inactivation to be performed with feedback from magnetic resonance imaging

## Introduction

Neuromodulation methods are widely used for perturbations of neural activity in both basic science and clinical practice, but monitoring the time course and spatial extent of modulatory tools in living subjects is challenging. Even with modern optogenetic and chemogenetic approaches^1,2^, measurement of actuator expression profiles is usually only possible postmortem, and the efficacy of these tools depends on light or drug level profiles that are rarely characterized. Traditional neuropharmacological perturbations are easier to apply in many species, but are also hard to monitor in vivo. Although fluorescent drug conjugates can be mapped histologically after death^3^, the resulting profiles may not reflect the distribution or dose that produced experimental neural activity perturbations of interest. Radiolabeled drugs can be mapped using nuclear imaging methods in living subjects^4^, but the tomographic instrumentation applied in such approaches typically provides poor spatiotemporal resolution, and synthesis and handling of the radiopharmaceuticals themselves is complex.

Here we describe an approach to image-guided manipulation of brain activity based on paramagnetic drugs that can be visualized noninvasively by magnetic resonance imaging (MRI). We apply the principle to muscimol, an agonist of γ-aminobutyric acid (GABA) A receptors that is widely used for targeted inactivation of neural structures, and that has previously been applied as a fluorescent conjugate for postmortem histological imaging^5^. By chemically conjugating muscimol to a gadolinium chelate, we sought to create a paramagnetic muscimol analog (ParaMus) whose distribution could be imaged in real time in vivo, while offering pharmacological properties comparable to muscimol itself.

## Results

### Synthesis and characterization of ParaMus

**Fig. 1:**
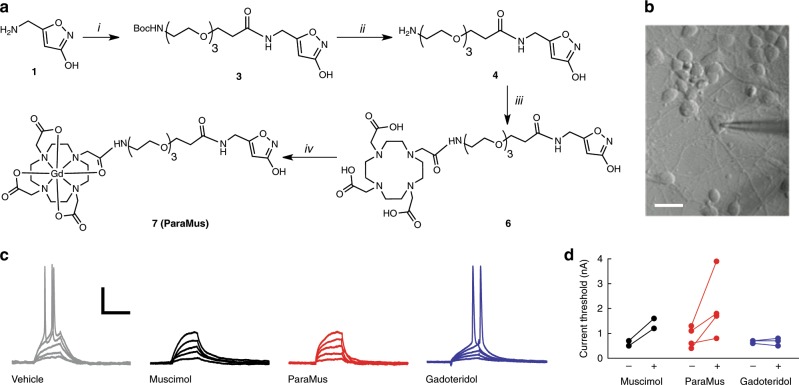
Synthesis and pharmacological characterization of ParaMus. **a** Chemical synthesis of ParaMus (**7**): (i) HOOC-PEG3-NHBoc, EDC/NHS, MOPS (100 mM, pH 5.5), (ii) TFA/DCM (80:20), (iii) DOTA-NHS, MOPS (25 mM, KCl 100 mM, pH 8), (iv) GdCl_3_, H_2_O, pH 5.5. **b** Setup for intracellular recording of primary rat cortical neurons (scale bar = 10 µm). **c** Representative current-clamp recordings in response to injection of 0.1-0.5 nA current injection in the presence of vehicle, 10 µM muscimol, 10 µM ParaMus, and 10 µM gadoteridol. Scale bars, horizontal = 20 ms, vertical = 10 mV. **d** Current thresholds for action potential generation before (−) and after (+) application of muscimol, ParaMus, or gadoteridol. Paired measurements are shown for two (muscimol), four (ParaMus), or three (gadoteridol) independent experiments.

The synthesis of ParaMus is diagrammed in Fig. 1a. The metal-free precursor to ParaMus (**6**) is prepared through cross-coupling of muscimol with the gadolinium binding ligand 1,4,7,10-tetraazacyclododecane-1,4,7,10-tetraacetic acid (DOTA), using a bifunctional triethylene glycol linker. ParaMus (**7**) is then formed by reacting **6** with excess of GdCl_3_ at pH 5. Integrity and purity of the final compound is indicated by mass spectrometry and liquid chromatography (Supplementary Fig. 1). The MRI potency of ParaMus is reflected by the slope of its effect on the reciprocal of the longitudinal relaxation time (*T*_1_) versus concentration, known as longitudinal relaxivity (*r*_1_). MRI measurements at 7 T and room temperature indicate that the *r*_1_ of ParaMus is 5.0 ± 0.2 mM^−1^ s^−1^ (error margins reflect SEM with *n* = 3, unless otherwise noted); this value is somewhat larger than the *r*_1_ value of 3.6 ± 0.3 mM^−1^ s^−1^ for gadoteridol^6^, a contrast agent that approximates the Gd-DOTA moiety of ParaMus, and indicates that conjugation to muscimol does not compromise contrast-inducing properties of the gadolinium complex (Supplementary Fig. 2). Like Gd-DOTA, ParaMus also displays a weak transverse relaxivity of 7.3 ± 0.6 mM^−1^ s^−1^ (Supplementary Fig. 3), which is unlikely to substantially alter image contrast except at high concentrations.

To assess the efficacy of ParaMus as an inhibitor of neuronal excitability, we examined its pharmacological activity in vitro. We subjected primary rat cortical neurons to intracellular current clamp (Fig. 1b), and measured current thresholds for action potential initiation under test and control conditions (Fig. 1c, d). We found that untreated neurons exhibit a current threshold of 0.9 ± 0.2 nA (*n* = 4), compared with 2.1 ± 0.6 nA following bath application of 10 µM ParaMus (*n* = 4). ParaMus also causes a decrease of 77 ± 17% in input resistance. Neurons treated with 10 µM muscimol also display an increase in current threshold, from 0.6 ± 0.1 to 1.4 ± 0.2 nA (*n* = 2), as well as a 71 ± 6% decrease in input resistance, similar to ParaMus. By contrast, gadoteridol elicits virtually no change in current threshold or input resistance when tested in the same assay. This indicates that the pharmacological effects of ParaMus arise from its muscimol component.

### Image-guided neural inactivation using ParaMus in vivo

**Fig. 2:**
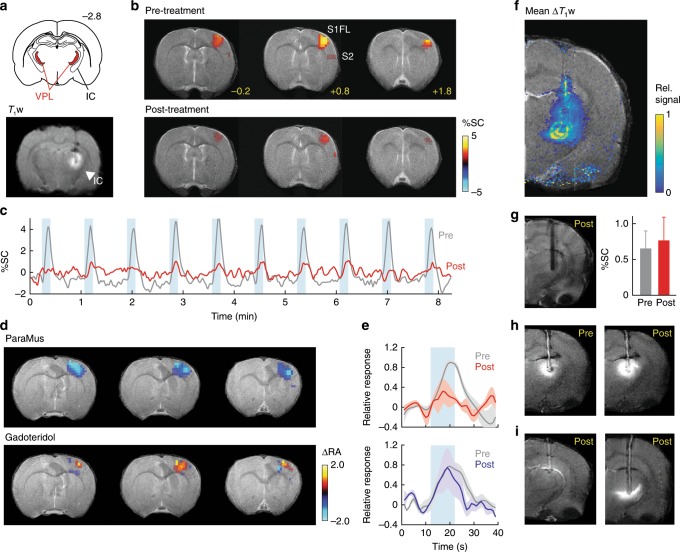
Image-guided manipulation of somatosensory responses in the rat brain. **a** Top: Coronal brain schematic^12^ at bregma = –2.8 mm, showing the location of VPL (red) bordered on the lateral side by the internal capsule (IC). Bottom: a corresponding *T*_1_-weighted (*T*_1_w) coronal image showing contrast enhancement in the VPL region following ParaMus infusion. Hypointense white matter IC signal denoted by arrowhead. **b** Forepaw stimulation-induced fMRI responses, in units of percent signal change (%SC), in a single animal before (top) and after (bottom) the ParaMus infusion shown in **a**. S1FL and S2 regions labeled; bregma coordinates in yellow. **c** Time course of fMRI signal in the S1FL region pre- (gray) and post-infusion (red), corresponding to data in **b**; stimulation blocks denoted by blue rectangles. **d** Change in fMRI response amplitudes (ΔRA), relative to pretreatment responses, after VPL-targeted ParaMus infusion (top, *n* = 5) or control infusion with gadoteridol (bottom, *n* = 4). **e** Mean relative peristimulus response time courses associated with the maps in **d**, pre- and post-treatment with ParaMus (top) or gadoteridol (bottom). Shading denotes SEM over five animals (top) or four animals (bottom). **f** Profile of mean relative *T*_1_-weighted MRI signal changes following ParaMus infusion in five animals. Control infusions lacking contrast agent produced no notable contrast enhancement. **g** A failed ParaMus infusion results in no enhancement in the posttreatment condition (left). Correspondingly, there is no decrease in fMRI response to forepaw stimulation after treatment (right); error bars denote SEM of responses over 10 stimulus cycles in a single animal. **h** Premature infusion of ParaMus is easily detectable by comparing pre- and post-treatment *T*_1_-weighted images. **i** Anomalous spatial distributions of ParaMus are recognized in images of two separate animals in the postinfusion condition.

To demonstrate image-guided manipulation of a neural system in vivo, we examined the ability of ParaMus to perturb brain responses to a sensory stimulus in anesthetized rats. We used functional MRI (fMRI) contrast to monitor brain-wide responses to electrical stimulation of the forepaw^7^ before, during, and after delivery of ParaMus to the ventral posterolateral nucleus of the thalamus (VPL), a relay point in the transmission of somatosensory input from the body to the cortex. The distribution of ParaMus infused via a cannula targeted to VPL could be visualized by *T*_1_-weighted MRI (Fig. 2a). Before this treatment, forepaw stimulation produced strong fMRI responses in the forelimb field of primary somatosensory cortex (S1FL), and weak responses in secondary somatosensory cortex (S2). After infusion of 1.5 µL ParaMus (1 mM) into the VPL region, responses were sharply reduced, as discernable from both activity maps (Fig. 2b) and response time courses (Fig. 2c). Group results (Fig. 2d, e) show that consistent decreases in the S1FL response to forepaw stimulation can be observed following VPL-targeted ParaMus treatment (*n* = 5), but not following control treatment with gadoteridol (*n* = 4). Mean response amplitudes are 69 ± 22% lower after ParaMus (significant with paired *t*-test *p* = 0.04, *n* = 5), but an average of only 3 ± 43% lower after gadoteridol (paired *t*-test *p* = 0.5, *n* = 4). These results indicate efficacy and specificity of ParaMus-dependent neural inactivation consistent with the neurophysiology of the somatosensory system.

The quintessential functionality afforded by ParaMus is an ability to relate differences in neuromodulator infusion profiles to resulting neurophysiological outcomes. In the experiments of Fig. 2d, e, ParaMus infusion covered a distribution of areas in and around VPL (Fig. 2f and Supplementary Fig. 4). Variations in the infusion profile among individuals could be detected by *T*_1_-weighted imaging, revealing the extent to which apparently equivalent injection procedures lead to different results. For instance, a failed infusion could be detected by the absence of *T*_1_ enhancement, as well as corresponding fMRI response changes (Fig. 2g). Premature infusion of ParaMus could be detected as *T*_1_ contrast or relaxation changes prior to purposeful injection (Fig. 2h and Supplementary Fig. 5). Anomalous infusion profiles arising from subtle differences in cannula placement or convection dynamics could also be recognized (Fig. 2i). *T*_1_-weighted MRI readouts therefore provide instant feedback on the characteristics of neuromodulatory drug delivery, with the potential to guide experimental strategies. Quantitative analysis of ParaMus-mediated *T*_1_ changes and corresponding effects in fMRI reveals a significant (correlation coefficient = 0.70, *p* = 0.04, *n* = 9) and approximately linear relationship between the extent of fMRI responses in S1FL and the percentage of sensory thalamus infused with ParaMus (Supplementary Fig. 6).

**Fig. 3:**
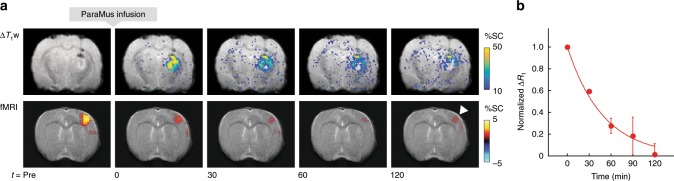
Time course of ParaMus-induced *T*1 contrast and fMRI response changes. **a** Maps showing infusion of contrast agent (top, bregma = –2.8 mm) and fMRI responses (bottom, bregma + 0.8 mm) as a function of time in the subject of Fig. 2b. Data are shown before (Pre) and after ParaMus infusion, as indicated by time points (*t*) in minutes shown below the images. The infusion maps display the change in *T*_1_-weighted signal (Δ*T*_1_w) due to ParaMus infusion, in units of percent signal change (%SC) compared with preinfusion contrast, overlaid on an anatomical image. The fMRI activation maps indicate mean response amplitudes induced by forepaw stimulation in 8.4-min trials beginning at each of the designated time points. Note the slight recovery of the S1FL response peak at the 120 min time point (arrowhead), after ParaMus contrast has largely dissipated, a result replicated also in a second animal. A small amount of ParaMus leakage is also detectable as Δ*T*_1_w hyperintensity around the needle in the Pre image. **b** Washout of ParaMus following infusion into brain, as reflected by the normalized change in *T*_1_ relaxation rate (*R*_1_) with respect to preinfusion conditions averaged over sensory thalamus. Values indicate mean and SEM (error bars) of data from two animals. The data are fit by an exponential decay curve (shown) with half-life 34 min.

Another key capability ParaMus provides is the possibility of relating drug distribution profiles at multiple time points to responses observed during and after infusion. In a representative animal, a decrease in fMRI responses to forepaw stimulation can be detected progressively throughout ParaMus infusion, followed by a partial recovery as the drug begins to wash out (Fig. 3a). Evaluation of *T*_1_ relaxation rate time courses in such experiments also enables in vivo estimation of the half-life for elimination of ParaMus from the brain (Fig. 3b and Supplementary Fig. 7), a value of ~30 min. Postmortem analysis of brain tissue after an imaging experiment permits identification of ParaMus by mass spectrometry and reveals that the contrast agent remains intact during the experimental period (Supplementary Fig. 8).

## Discussion

These results demonstrate that ParaMus combines the pharmacological properties of muscimol with the MRI properties of commercial contrast agents, enabling imaging-based assessment and control over neural manipulations in the living brain. The reagent is straightforward to produce and apply, and could immediately be used in a range of biomedical contexts and in species ranging from rodents to primates, possibly including human subjects. In addition to fMRI responses like those examined here, other physiological and behavioral measures could also be investigated in conjunction with ParaMus-mediated perturbations, as long as MRI mapping of the drug distribution is performed soon before or after the relevant experiments. Non-MRI activity measurements are most feasible in the immediate vicinity of ParaMus infusion, where fMRI signal might be distorted by the contrast agent’s magnetic susceptibility. The closest alternative to ParaMus-based procedures introduced here is afforded by mixing muscimol with conventional MRI contrast agents like gadoteridol^8^, but the properties of such admixtures are considerably inferior. Muscimol and gadoteridol differ in molecular weight by a factor of five, and while muscimol is positively charged at pH 7, gadoteridol is neutral. Diffusion, molecular adhesion, cell uptake, and pharmacokinetics of the two compounds are likely to differ substantially, raising interpretive questions that are avoided when using ParaMus.

In future work, the idea of conjugating paramagnetic moieties to neuromodulatory agents could be extended to create additional MRI-detectable drugs. Construction of ParaMus exploits the fact that modification of the muscimol amine does not compromise pharmacological activity, but additional neurotransmitter receptor ligands also possess sites where modification is possible without abrogating receptor affinity^9–11^. A toolkit of imageable drugs could potentially be multiplexed by attaching different types of contrast agent—for instance employing *T*_1_ vs. transverse relaxation (*T*_2_) or chemical exchange saturation transfer contrast-inducing moieties—to different neuromodulatory substances. The approach presented here may therefore promote an unprecedented level of informed, multimodal control over neural function in deep tissue.

## Methods

### Reagents and general chemical methods

All solvents were of reagent grade and all other materials were purchased and used as received. Chemicals were procured from Sigma Aldrich (St. Louis, MO) unless otherwise noted. Compound **5** was procured from Macrocyclics (Plano, TX). Silica gel (230-400 mesh, VWR, Radnor, PA) and octadecyl-functionalized silica gel (RP-18, Waters, Milford, MA) were used for column chromatography. Analytical thin-layer chromatography was performed by using Merck 60 F254 silica gel (precoated aluminum sheets, 0.25 mm thick). Reverse phase preparative high-performance liquid chromatography (HPLC) traces were recorded at 298 K using a Waters (Milford, MA) HPLC system equipped with a semi-preparative C18 column. A gradient elution with a solvent system composed of water/acetonitrile with 0.1% trifluoroacetic acid (TFA) was applied for a total run time of 33 min.

### Mass spectrometry

Both standard and high-resolution electrospray mass spectrometry were recorded on an Agilent Technologies (Santa Clara, CA) HP 8453 spectrometer, operating in positive or negative ion mode as stated, with MeOH as the carrier solvent. Matrix-assisted laser desorption ionization time of flight (MALDI-TOF) mass spectra were recorded on a Bruker Instruments (Ettlingen, Germany) MicroFlex instrument with α-cyano-4-hydroxycinnamic acid employed as the matrix and 50/50 water/acetonitrile + 0.1% TFA used to prepare all samples.

### Elemental analysis

Absolute concentrations of final purified complexes for in vitro and in vivo studies were calculated by gadolinium content using inductively coupled plasma mass spectrometry on an Agilent 7900 ICP-MS instrument. Complexes were digested in concentrated nitric acid at 70 °C for 2 h, before being diluted into 2% nitric acid at a working gadolinium concentration of 10–100 ppb. Gadolinium concentration was calibrated with nine data points across the range of 1–500 ppb using known stock concentrations. Overall, 10 ppb erbium was used in all samples as an internal standard. Each sample was prepared in triplicate and each data point was measured in duplicate.

### NMR spectroscopy

^1^H and ^13^C NMR spectra of each compound were recorded in commercially available deuterated solvents on a Bruker Avance III DPX 400 (^1^H at 400 MHz, ^13^C at 101 MHz). All chemical shifts are given in ppm and coupling constants are in Hz.

### Synthetic methods

Further detailed information regarding the synthesis and characterization of Paramus (**7**) and related compounds are reported in Supplementary Methods.

### In vitro MRI

MRI data were acquired in a 12 cm outer diameter birdcage transceiver for imaging at room temperature in a 20 cm bore Bruker 7 T Avance III MRI scanner. Samples at varying concentrations in 20 mM 4-(2-hydroxyethyl)-1-piperazineethanesulfonic acid (HEPES) buffer, pH 7.4, were loaded into a 384-well clear polystyrene plate (Thermo Fisher Scientific, Waltham, MA), which had been pre-cut in half to optimally fit the coil. Unused wells were filled with buffer. Overall, 2 mm slices were imaged through the samples with a field of view (FOV) of 5 × 5 cm and data matrix of 256 × 256 points. Data for longitudinal (*r*_1_) and transverse (*r*_2_) relaxivity measurements were acquired using a multi-slice multi-echo pulse sequence (echo time, *TE* = 12–360 ms, repetition time, *TR* = 100, 200, 300, 400, 500, 600, 700, 800, 900, 1000, 1200, 1500, 3000, 5000 ms). Custom routines written in MATLAB (Mathworks, Natick, MA) were used to reconstruct the images and compute relaxation time constants by fitting image intensity data to exponential decay curves. Relaxivity values were then computed from the slope of *T*_1_ and *T*_2_ relaxation rates versus contrast agent concentration, as determined by ICP-MS. Values of *r*_1_ = 5.0 ± 0.2 mM^−1^ s^−1^ and *r*_2_ = 7.3 ± 0.6 mM^−1^ s^−1^ were obtained for ParaMus, where the error margins represent SEM of *n* = 3 measurements.

### Animals

All animal procedures were performed in strict compliance with US Federal guidelines, with oversight by the MIT Committee on Animal Care. A total of nine male Sprague-Dawley rats (300–400 g), purchased from Charles River Laboratories (Wilmington, MA), were used for the in vivo data presented in this paper. The animals were housed and maintained on a 12 h light/dark cycle with ad libitum access to food and water. Sample sizes for animal experiments were chosen to ensure reproducibility and quantify observed effects, rather than to guarantee recognition of prespecified effect sizes with a given level of power. Experiments were not randomized or blinded.

### Electrophysiology

Cortical neurons from E18 Sprague-Dawley rat embryos were kindly provided by the laboratory of Elly Nedivi (Picower Institute for Learning and Memory). The cells were seeded at 1.5 × 10^5^ cells per 18 mm on #1 round coverslips coated with laminin (Life Technologies, Woburn, MA) and poly-D-lysine (Sigma-Aldrich, St. Louis, MO). Prior to electrophysiological measurements, cells were incubated in 2% B-27 supplement and 1% GlutaMAX (Thermo Fisher Scientific) for seven days. Recordings were performed at room temperature from neurons in extracellular solution containing 145 mM NaCl, 5 mM KCl, 1 mM MgCl_2_, 1 mM CaCl_2_, 5 mM HEPES, 5 mM glucose, 20 mM sucrose, 0.25 mg/L phenol red, and 10 µM D-serine (all materials from Sigma-Aldrich). The pH of this solution was adjusted to 7.4 with NaOH, and its osmolarity was adjusted to 315 mOsms with sucrose. Electrodes were pulled in two stages from borosilicate glass capillaries (A-M systems, Sequim, WA) using a horizontal pipette puller (PD-97, Sutter instruments, Novato, CA), resulting in resistances of 8–12 MΩ when filled with an internal solution containing 145 mM KCl, 10 mM HEPES, 5 mM adenosine triphosphosphate, 0.2 mM guanosine triphosphate, and 10 mM ethylene glycol-bis(β-aminoethyl ether)-*N*,*N*,*N*′,*N*′-tetraacetic acid, adjusted to pH 7.2 with KOH. Whole-cell current-clamp recordings were performed using AxoClamp 1B amplifier (Axon Instruments, Union City, CA). Cell membrane potentials were initially set to –60 mV, and voltage response to 50 ms current pulses of increasing amplitude, in 0.1 nA steps, were used to assess action potential thresholds and input resistances. To determine the effects of pharmacological agents, this procedure was performed following bath application of 10 µM muscimol (Sigma-Aldrich), 10 µM gadoteridol (Sigma-Aldrich), or 10 µM ParaMus.

### Surgical methods

In preparation for imaging experiments with intracranially-infused ParaMus or control agents, rats were implanted with infusion cannulae targeting VPL. Animals were anaesthetized with 2% isoflurane, shaved, and mounted in a rodent stereotaxic device (David Kopf Instruments, Tujunga, CA). Heart rates and blood oxygenation levels were monitored by a pulse oximeter (Nonin Medical, Plymouth, MN). Each rat was given a subcutaneous injection of slow-release buprenorphine (MIT pharmacy) at a dose of 1 mg/kg for analgesia at the beginning of surgery. The scalp was retracted and 28 G holes were drilled into the skull 3.2 mm posterior and 3 mm lateral to bregma, unilaterally or bilaterally, with coordinates based on a standard rat brain atlas^12^. Overall, 28 G cannula guides (Plastics One, Roanoke, VA) designed to project 2 mm below the surface of the skull were implanted and secured using C&B Metabond dental cement (Parkell, Edgewood, NY). Small plastic head-posts were implanted to facilitate head fixation during the MRI experiments. Finally, 32 G dummy cannulae designed to fit the guide cannulae were inserted to protect the openings when not in use. Rats were allowed to recover from surgery for at least three days before imaging.

### In vivo MRI

Immediately prior to imaging experiments, cannula-implanted rats were briefly anesthetized using 3% isoflurane and maintained at 2% isoflurane during preparation. Animals were intubated and ventilated, and an intraperitoneal catheter was established for drug delivery. Animals were then placed onto a cradle and secured in place via screws that attached to the implanted head-post. Isoflurane was discontinued and intraperitoneal bolus doses of 0.1 mg/kg dexdomitor (MIT pharmacy) and 1 mg/kg pancuronium (Sigma-Aldrich) were administered, followed by continuous delivery of 0.2 mg/kg/h dexdomitor and 2 mg/kg/h pancuronium thereafter. Respiration, heart rate, and blood oxygen saturation were monitored, and temperature was maintained with a circulating warm water pad (Gaymar, Orchard Park, NY) for the remainder of the procedures. Overall, 32 G internal cannula (Plastics One) preloaded with 1 mM ParaMus, 1 mM gadoteridol, or saline vehicle were inserted at this time, targeting VPL via the previously implanted guide cannulae. Internal cannulae extended 5–6 mm below the brain surface. Animals were then inserted into the MRI scanner.

In vivo imaging was performed using a 9.4 T Biospec MRI scanner (Bruker) scanner operating with a cross coil volume transmit, surface receive configuration. A rapid acquisition with refocused echoes (RARE) pulse sequence were used to acquire *T*_2_-weighted anatomical images, with number of averages (NA) = 4, matrix size = 256 × 192, FOV = 2.56 cm × 1.28 cm, slice thickness = 1 mm, *TR* = 5000 ms, effective *TE* = 30 ms, and RARE factor = 8. To quantify the extent of ParaMus infusion, *T*_1_-weighted RARE images were also acquired, using NA = 6, matrix size = 128 × 64, FOV = 2.56 cm × 1.28 cm, slice thickness = 1 mm, *TR* = 252 ms, and effective *TE* = 5 ms. For *T*_1_ mapping, data were acquired with additional *TR* values of 429, 600, 900, 1000, 1500, 2000, 2500, and 3000 ms. For functional imaging, echo-planar imaging (EPI) image series were acquired during alternating blocks of forepaw stimulation and rest. EPI scan parameters were NA = 1, matrix size = 64 × 32, FOV = 2.56 cm × 1.28 cm, slice thickness = 1 mm, *TR* = 2000 ms, and effective *TE* = 16 ms.

Forepaw stimulation itself was performed using 9 Hz pulse trains with 1 ms pulse width and current of 3–6 mA. Forepaw stimulation blocks were 10 s long, and were delivered in ten cycles with 40 s rest periods in between. In two animals, forepaw imaging trials were performed repeatedly, every 30 min, along with *T*_1_ mapping to determine the time course of ParaMus washout and corresponding changes in fMRI responses. In seven animals, measurement of fMRI responses before vs. after ParaMus infusion was performed in parallel with measurement of fMRI responses before vs. after control treatment with saline or gadoteridol. In these cases, test and control solutions were delivered via bilateral cannulae inserted in opposite hemispheres, and corresponding contralateral forepaws were stimulated in each case. Four animals that experienced suboptimal ParaMus targeting were excluded from the group data quantifying the efficacy of VPL ParaMus delivery (Fig. 2d), but were included in Supplementary Fig. 6, which specifically relates variation in ParaMus delivery to differences in the effects on fMRI responses.

### MRI data analysis

Images were reconstructed using Paravision software (Bruker) and further analyzed using custom routines implemented in MATLAB. Preprocessing of functional imaging data was performed using the AFNI software package (National Institute of Mental Health, Bethesda, MD)^13^. Steps included motion correction using a rigid-body volume registration algorithm, spatial and temporal smoothing, voxel-wise intensity normalization, and segmentation of brain from nonbrain voxels. To coregister images into a uniform space, a reference anatomical scan was first manually aligned to a digitized version of a standard rat atlas^12^. All other anatomical scans were then aligned to the reference image using a nine-parameter affine transformation implemented in AFNI. Each animal’s EPI scans were aligned to the corresponding coregistered anatomical scan. The time-series data from the EPI scans were smoothed with a Gaussian spatial kernel of 1 mm full width at half-maximum prior to statistical analysis, and each voxel time course was subsequently temporally smoothed using a sliding box window of width 3.

Initial statistical analysis of preprocessed EPI scans was performed in AFNI. Regression coefficients for activation maps were computed by convolving the electrical forepaw stimulus times with a hemodynamic response model. Six motion correction parameters were included from each animal as nuisance regressors. Outlier scans detected by median absolute deviation from time-series trends in each data set were censored from the analysis.

Response amplitudes and *t* statistics for voxels that fell within the atlas-defined somatosensory cortex^12^ were used as input for further analysis of fMRI results in MATLAB. To generate response maps such as Fig. 2b, the response in units of percent signal change (%SC) was indicated for all voxels whose *p* value was below an uncorrected significance threshold of 0.05. To generate time courses in Fig. 2c, responses were averaged over all voxels meeting the significance criterion prior to ParaMus infusion. To generate the difference maps in Fig. 2d, the percent signal change estimated by general linear modeling to a stimulus regressor before injection was subtracted from the percent signal change observed after injection on a voxel-by-voxel basis. To compensate for variability in the pretreatment response amplitudes among animals, difference maps from each animal were normalized to the peak values observed before treatment and then averaged across the animals. Thus, blue corresponding to a value of –1 indicates complete abrogation of the response on average, as a result of treatment. The time courses of Fig. 2e were computed by similarly normalizing each time course to the peak value observed in the preinjection condition, followed by averaging over voxels in S1FL and then over animals. Error margins reported in each case represent the standard error of the mean over animals (*n* = 5 for ParaMus treatment and *n* = 4 for gadoteridol control treatment).

Maps of contrast agent-dependent signal change in *T*_1_-weighted MRI scans, such as Fig. 2f, were computed on by applying the formula %SC = 100 × (*S*_*post*_ – *S*_*pre*_)/*S*_*pre*_ on a voxel-wise basis, where *S*_*pre*_ and *S*_*post*_ are the signal amplitudes observed before and after infusion, respectively. These maps were averaged over animals (*n* = 5). Maps of *T*_1_ and *R*_1_ (= 1/*T*_1_) were computed by exponential fitting to *T*_1_-weighted data acquired at multiple *TR* values, as specified above. Values were averaged across sensory thalamus (VPL and ventroposterior thalamus, a total of 40 voxels) to obtain reported means. Correspondence of ParaMus injection spread and fMRI activation extent was examined by computing the percentage of sensory thalamus filled during ParaMus infusion, and plotting this against the percent change in the number of voxels showing significant (*F*-test *p* < 0.05) stimulus-dependent modulation after vs. before infusion.

### Analysis of ParaMus in injected brain tissue

A rat was sacrificed and purfused with NaCl solution (0.9%) ~1 h after ParaMus infusion as described above. The collected brain sample was dounced in methanol (5 mL) for 5 min and centrifuged at 3000 × *g* for 10 min. The supernatant was collected and the residue was dounced again in methanol for 1 min and centrifuged for another 10 min at 3000 × *g*. This procedure was repeated two further times. All collected methanol supernatant fractions were pooled and filtered through a 0.2 µm filter. The resulting clear solution was concentrated to dryness under vacuum, dissolved in 500 µL deionized water, and filtered through a 3 kDa cutoff filter. The filtrate was then concentrated to 250 µL under vacuum and analyzed using an Agilent 6125B mass spectrometer attached to an Agilent 1260 Infinity LC system.

### Reporting Summary

Further information on research design is available in the Nature Research Reporting Summary linked to this article.

## Supplementary information

Supplementary Information

Reporting Summary

## Data Availability

Scripts used for data analysis are available upon reasonable request.

## Code availability

Raw MRI datasets generated during and/or analyzed during the current study are available from the corresponding author on reasonable request.

## References

[1] Boyden ES, Zhang F, Bamberg E, Nagel G, Deisseroth K (2005). Millisecond-timescale, genetically targeted optical control of neural activity. Nat. Neurosci..

[2] Alexander GM (2009). Remote control of neuronal activity in transgenic mice expressing evolved G protein-coupled receptors. Neuron.

[3] Daly CJ, McGrath JC (2003). Fluorescent ligands, antibodies, and proteins for the study of receptors. Pharm. Ther..

[4] Dierckx, R. A. J. O. et al. (eds.) *PET and SPECT of Neurobiological Systems*. (Springer-Verlag, Berlin, 2014).

[5] Wang H, Standifer KM, Sherry DM (2000). GABA(A) receptor binding and localization in the tiger salamander retina. Vis. Neurosci..

[6] Tweedle MF (1997). The ProHance story: the making of a novel MRI contrast agent. Eur. Radio..

[7] Hyder F, Behar KL, Martin MA, Blamire AM, Shulman RG (1994). Dynamic magnetic resonance imaging of the rat brain during forepaw stimulation. J. Cereb. Blood Flow. Metab..

[8] Wilke M, Turchi J, Smith K, Mishkin M, Leopold DA (2010). Pulvinar inactivation disrupts selection of movement plans. J. Neurosci..

[9] Bartels E, Wassermann NH, Erlanger BF (1971). Photochromic activators of the acetylcholine receptor. Proc. Natl Acad. Sci. USA.

[10] Volgraf M (2006). Allosteric control of an ionotropic glutamate receptor with an optical switch. Nat. Chem. Biol..

[11] Donthamsetti PC (2017). Optical control of dopamine receptors using a photoswitchable tethered inverse agonist. J. Am. Chem. Soc..

[12] Paxinos, G. & Watson, C. *The Rat Brain in Stereotaxic Coordinates, Compact 6th Ed.* (Academic Press, Boston, 2009).

[13] Cox RW (1996). AFNI: software for analysis and visualization of functional magnetic resonance neuroimages. Comput. Biomed. Res..

