# Supplementary material for: Image-guided neural activity manipulation with a paramagnetic drug
Source: Nat Commun. 2020 Jan 9;11:136. doi: 10.1038/s41467-019-13933-5 (PMC6952355; doi:10.1038/s41467-019-13933-5)

Image-guided neural activity manipulation with a paramagnetic drug

Bricault, Barandov *et al.*

Supplementary Information

SUPPLEMENTARY FIGURES

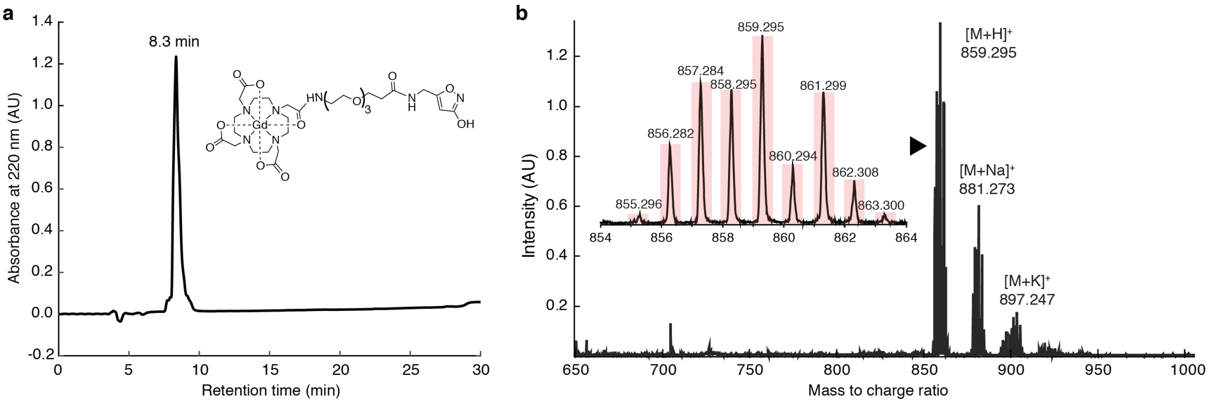

Supplementary Figure 1. Mass and purity of ParaMus. (a) HPLC trace demonstrating retention time (8.3 min) and purity of ParaMus (inset), as indicated by absorbance at 220 nm. **(b)** Matrix assisted laser desorption ionization time of flight (MALDI-TOF) mass spectrum of ParaMus. The inset magnifies the peak associated with the presumed $[M^{ParaMus}+H]^+$ species, and the light red boxes indicate the theoretical isotopic pattern, confirming identity of the compound.

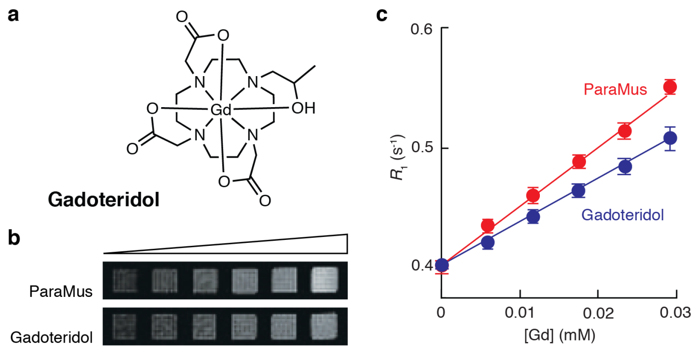

Supplementary Figure 2. Longitudinal relaxivity of ParaMus and gadoteridol. (a) Structure of gadoteridol, which approximates the neutral gadolinium DOTA moiety of ParaMus. **(b)** Images of successive concentrations of ParaMus and gadoteridol (0-30 µM) imaged using T_1-weighted MRI at 7 T and room temperature in 100 mM HEPES, pH 7.4. **(c)** T_1-weighted MRI data were used to compute longitudinal relaxation rates (R_1) for ParaMus (red) and gadoteridol (blue) solutions, as a function of concentration. Error bars indicate SD of three measurements each. Slopes of these datasets give estimated r_1 values of 5.0 ± 0.2 mM^{-1}s^{-1} for ParaMus and 3.6 ± 0.3 mM^{-1}s^{-1} for gadoteridol.

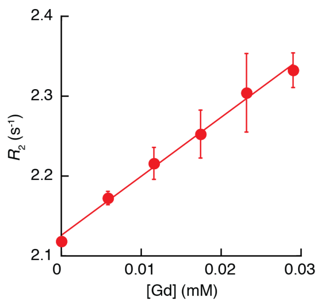

Supplementary Figure 3. Transverse relaxivity of ParaMus. Values of the transverse relaxation rate (R_2) are shown as a function of ParaMus at the gadolinium concentrations shown, measured by MRI at 7 T and room temperature in 100 mM HEPES, pH 7.4. Values indicate mean ± SD of three measurements each.

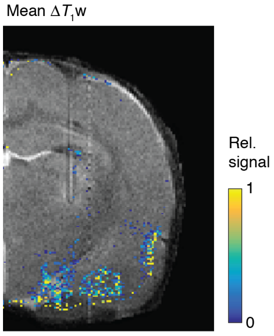

Supplementary Figure 4. Contrast enhancement due to VPL-targeted vehicle injection. A map of mean relative T_1-weighted MRI signal changes following saline vehicle infusion in two animals, indicating that contrast changes observed in the analogous data of Fig. 2f require the presence of ParaMus.

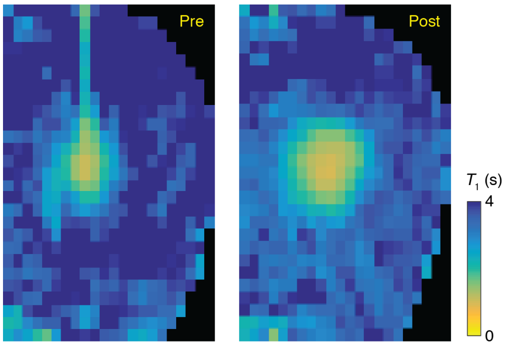

Supplementary Figure 5. T_1 maps indicating pre-infusion leakage of ParaMus. Longitudinal relaxation time (T_1) maps were obtained before (Pre, left) and after (Post, right) infusion of ParaMus in the animal subject of Fig. 2h. Low T_1 values in the center of the field of view in both Pre and Post conditions indicate the presence of contrast agent, corresponding to brightening seen in the analogous T_1-weighted images.

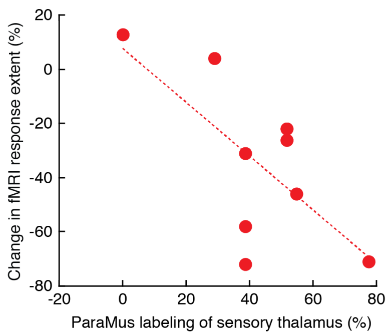

Supplementary Figure 6. Correspondence between ParaMus delivery and fMRI effects. The extent of ParaMus infusion was estimated by computing the percentage of voxels in sensory thalamus that experienced an R_1 change over 20% during ParaMus infusion. The change in fMRI response was determined as the percent change in the number of voxels showing significant (F-test $p < 0.05$) stimulus-dependent modulation after *vs.* before ParaMus infusion. The dashed line indicates the least-squares linear fit, representing a significant correlation across nine animals with correlation coefficient = 0.70 and $p = 0.04$.

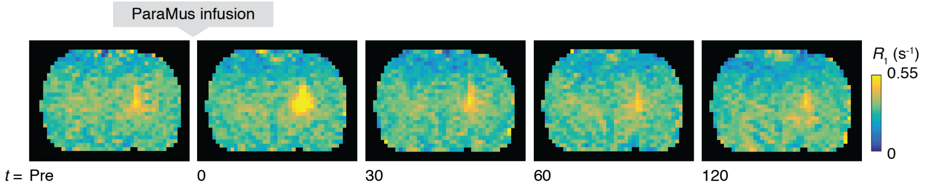

Supplementary Figure 7. Time course of ParaMus-induced T_1 relaxation changes. Coronal maps of the T_1 relaxation rate (R_1) throughout the brain slice in which ParaMus infusion was performed in the animal subject of Figure 3a (bregma = –2.8 mm). Data represent values both prior to (Pre) and at time points indicated in minutes after ParaMus infusion.

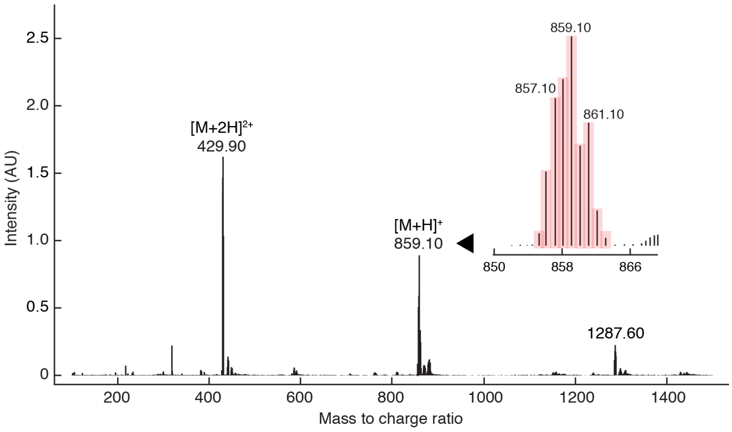

Supplementary Figure 8. Analysis of ParaMus in injected brain tissue. Brain tissue from a ParaMus-infused rat brain was homogenized and subjected to liquid chromatography mass spectroscopic analysis. Intact ParaMus was clearly recognizable from mass peaks corresponding to the $[M+H]^+$ species and $[M+2H]^{2+}$ species. Inset magnifies the peak associated with the $[M+H]^+$ species, highlighting correspondence of the theoretical isotopic pattern (pink boxes) with the observed peaks in black.

SUPPLEMENTARY METHODS

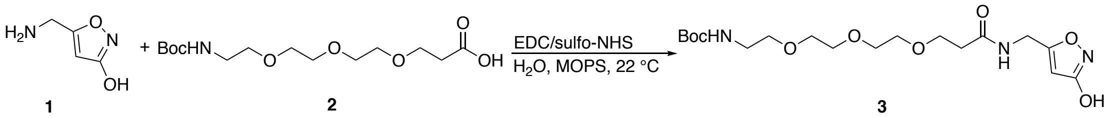

NHBoc-PEG3-COOH Mus-PEG3-NHBoc

Mus-PEG3-NHBoc, 3. NHBoc-PEG3-COOH (**2**) (141 mg, 0.44 mmol) was dissolved in 3-(*N*-morpholino)propanesulfonic acid (MOPS) buffer (100 mM, pH 5.5) and 1-ethyl-3-(3-dimethylaminopropyl)carbodiimide (EDC) (168 mg, 0.88 mmol) and N-hydroxysulfosuccinimide (sulfo-NHS) (280 mg, 1.32 mmol) were added. The reaction solution was stirred at room temperature for 5 min and muscimol (**1**) (50 mg, 0.44 mmol) was added; the resulting clear solution was stirred at ambient temperature for 18 h. The reaction mixture was purified by preparative high performance liquid chromatography (HPLC) [silica-C18, eluent gradient H_2O:acetonitrile (MeCN) from 95:5 to 10:90]. Yield: 95 mg (68%). High resolution mass spectrometry (HRMS) with negative-mode electrospray ionization (ESI-) *m/z* calc. 416.203 for $C_{18}H_{30}N_3O_8^-$ found 416.211 $[M-H]^-$. ^1H nuclear magnetic resonance (NMR) (400 MHz, Methanol-d_4) δ 5.54 (s, 1H), 4.06 (s, 2H), 3.67 – 3.39 (m, 4H), 3.28 (d, J = 5.8 Hz, 4H), 3.22 – 3.15 (m, 4H), 2.91 (td, J = 5.6, 1.9 Hz, 4H), 2.60 (s, 1H), 2.24 (t, J = 6.3 Hz, 1H), 2.19 (t, J = 6.0 Hz, 3H), 1.12 (s, 9H). ^{13}C NMR (101 MHz, Methanol-d_4) δ 175.23, 174.11, 171.99, 171.86, 94.51, 80.06, 70.99, 68.09, 67.75, 56.35, 43.57, 41.23, 37.46, 36.50, 35.75, 28.76.

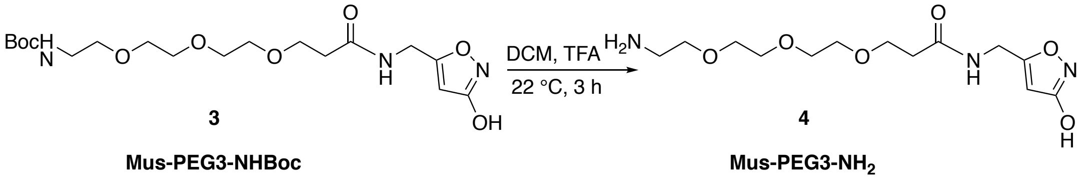

Mus-PEG3-NHBoc Mus-PEG3-NH2

Mus-PEG3-NH2, 4. Compound **3** (60 mg) was suspended in dichloromethane (3 mL) and trifluoroacetic acid (3 mL) was added. The resulting solution was stirred at room temperature for 3 h. After removal of all volatiles in vacuum the resulting residue was dissolved in deionized water (3 mL) and freeze-dried to afford **4** as colorless oil. Yield: 38 mg (95%). HRMS (ESI+) *m/z* calc. 318.175 for $C_{13}H_{24}N_3O_6^+$, found 318.166 $[M+H]^+$. ^1H NMR (400 MHz, Methanol-d4) δ 5.85 (s, 1H), 4.37 (s, 2H), 3.76 (t, J = 6.0 Hz, 3H), 3.70 (t, J = 5.0 Hz, 2H), 3.66 (s, 4H), 3.64 – 3.61 (m, 7H), 3.12 (t, J = 5.1 Hz, 2H), 2.51 (t, J = 6.0 Hz, 3H). ^{13}C NMR (101 MHz, Methanol-d_4) δ 174.14, 172.07, 171.85, 94.56, 71.51, 71.37, 71.18, 71.17, 68.08, 67.82, 40.63, 37.30, 36.47.

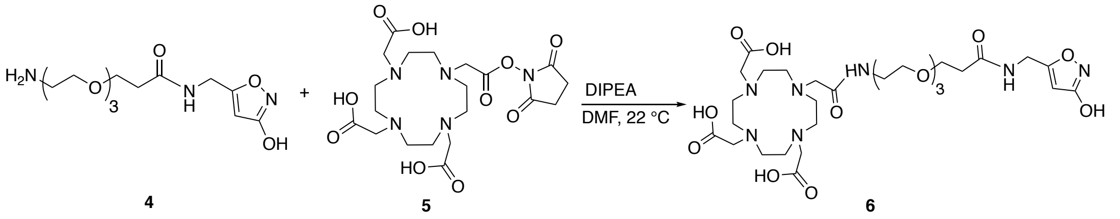

DOTA-PEG-Mus

DOTA-PEG-Mus, 6. Compound **4** (30 mg, 0.09 mmol) and diisopropylethylamine (13 mg, 0.1 mmol) were dissolved in anhydrous dimethylformamide (3 mL) and **5** (68 mg, 0.09 mmol) was added. The reaction solution was stirred at room temperature for 20 h and after removal of all volatiles purified by HPLC (C18 coated silica, H_2O [0.1%v trifluoroacetic acid (TFA)]:MeCN (0.1%v TFA), 95:5 to 5:95 in 32 min, R_t = 8.3 min). Yield: 60 mg (85%). HRMS (ESI-) *m/z* calc. 702.331 for $C_{29}H_{48}N_7O_{13}^-$, found 702.314 $[M-H]^-$. ^1H NMR (400 MHz, Methanol-d^4) δ 7.92-7.76 (m, 1H), 7.6-7.5 (m, 1H), 4.39 (s, 2H), 3.88-3.76 (m,10H), 3.64 (s, 8H), 3.58 (t, 2H, J_{HH} = 4 Hz), 2.52 (t, 2H, J_{HH} = 4 Hz). 13C NMR (101 MHz, Methanol-d^4) δ 172.72,

170.68, 170.56, 127.00, 125.85, 117.25, 110.02, 93.16, 70.14, 70.02, 69.84, 69.68, 68.90, 66.68, 54.43, 42.38, 38.97, 36.00, 35.14, 27.02, 17.30, 15.86.

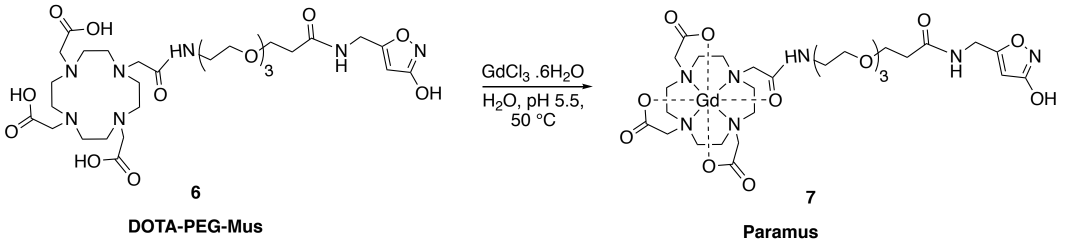

Paramus, 6. Gd(III)$Cl_3.6H_2O$ (6.3 mg, 0.017 mmol) was added to a stirred solution of 5 (10.0 mg, 0.014 mmol) in water (2 mL) and the pH was adjusted to 5.5 using $NaOH_{(aq)}$. The reaction was left stirring overnight at 50 °C. After cooling to rt, the pH was increased to 10 and the precipitated excess metal hydroxide was removed by centrifugation. The solution was adjusted back to pH 7 with $HCl_{(aq)}$ and lyophilized overnight. The resulting white solid was purified by preparative HPLC (C18 coated silica, H_2O (0.1%v TFA):MeCN (0.1%v TFA), 95:5 to 5:95 in 32 min, retention time = 8.4 min). Yield: 6.5 mg (54%). MALDI-TOF *m/z* calc. 859.24729 for $C_{29}H_{47}GdN_7O_{13}$, found 859.29536 $[M+H]^+$.

^{1}H NMR spectrum of Mus-PEG3-NHBoc

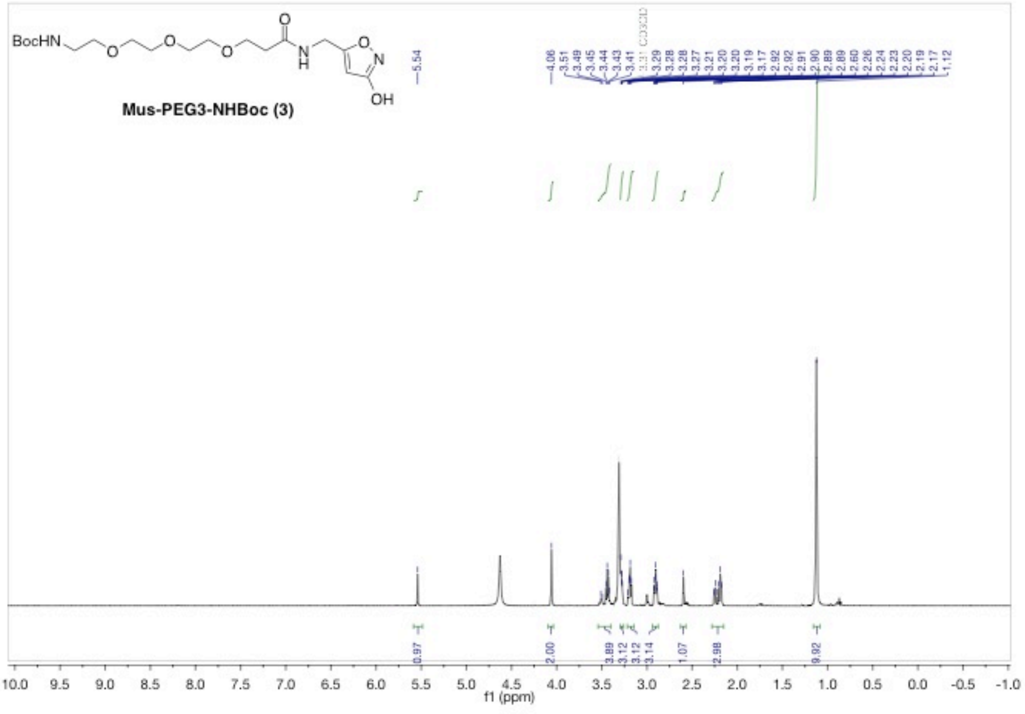

^{13}C NMR spectrum of Mus-PEG3-NHBoc

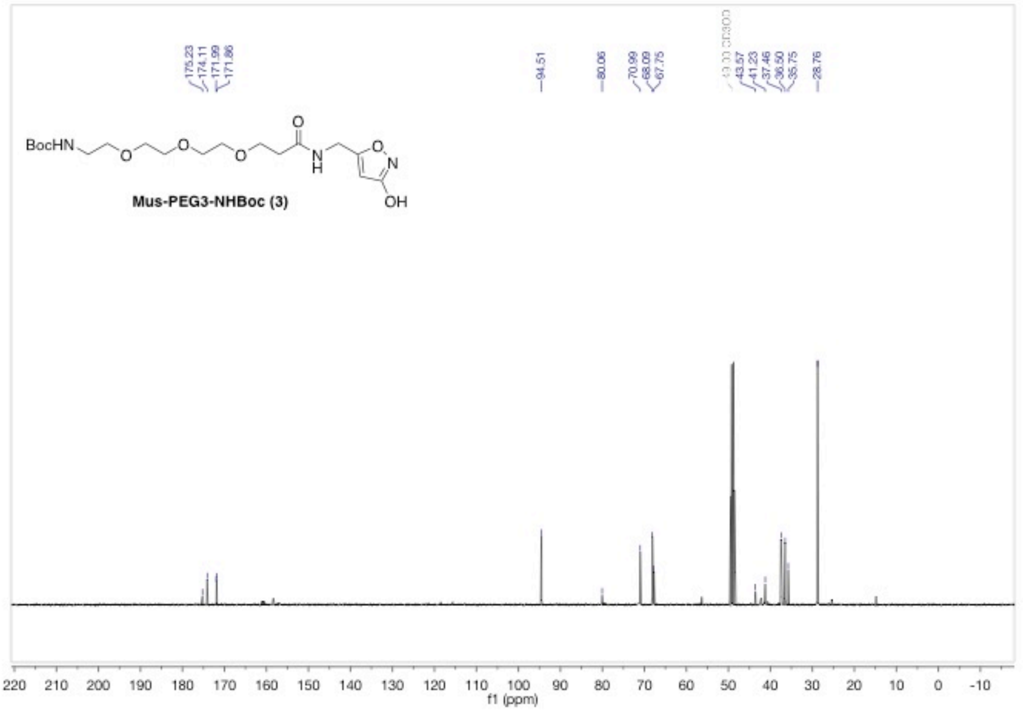

^{1}H NMR spectrum of Mus-PEG3-NH$_2$

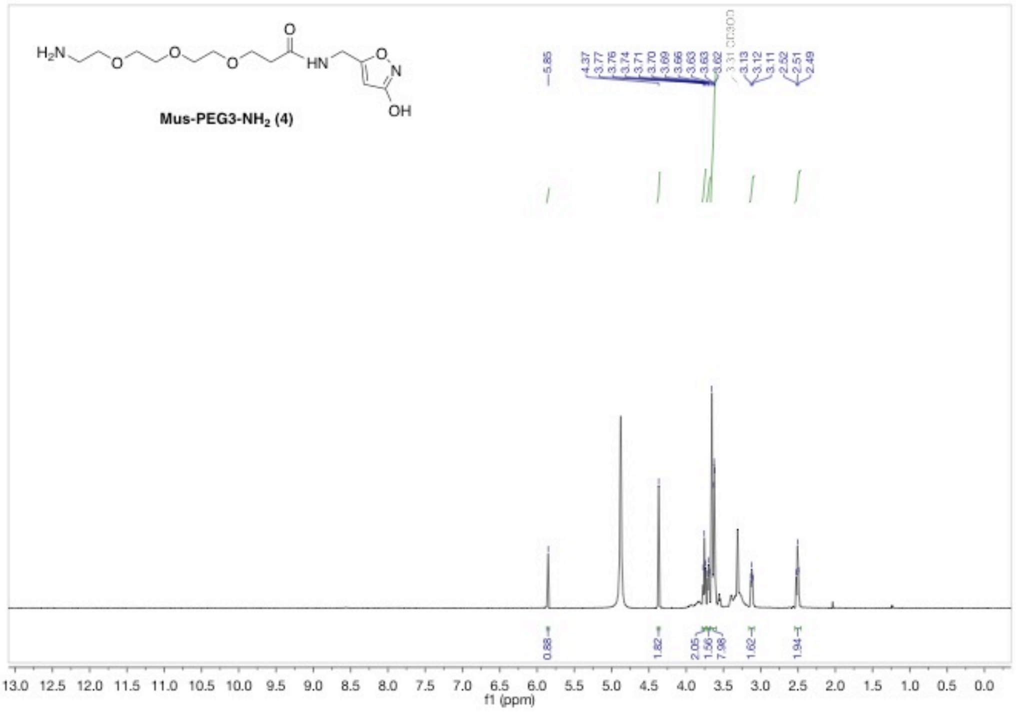

^{13}C NMR spectrum of Mus-PEG3-NH$_2$

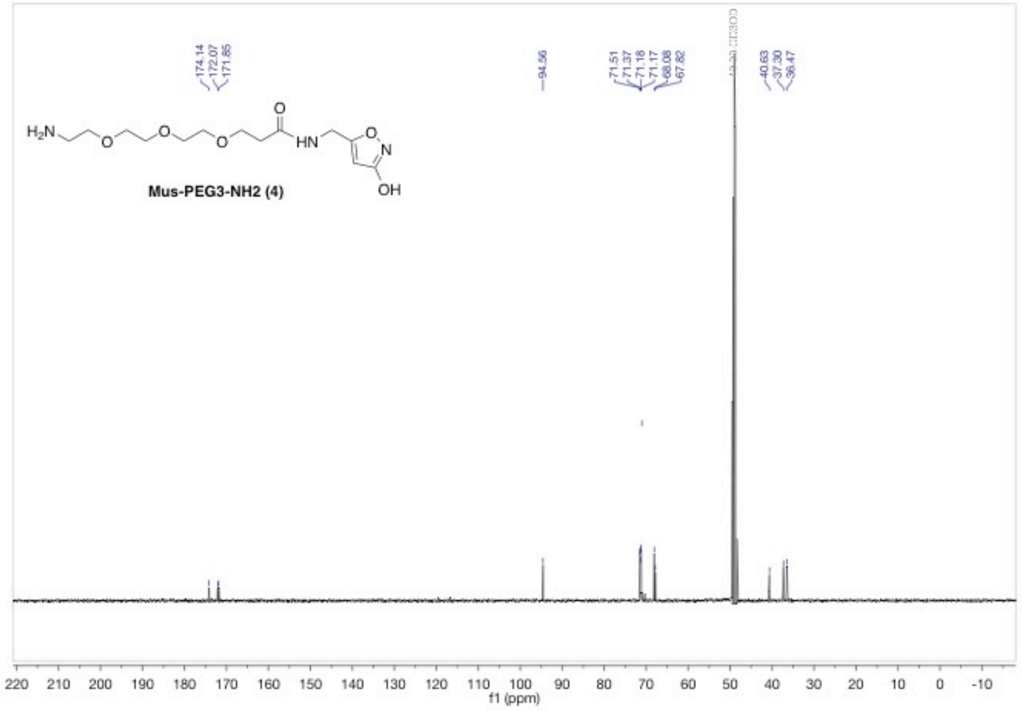

^{1}H NMR spectrum of DOTA-PEG-Mus

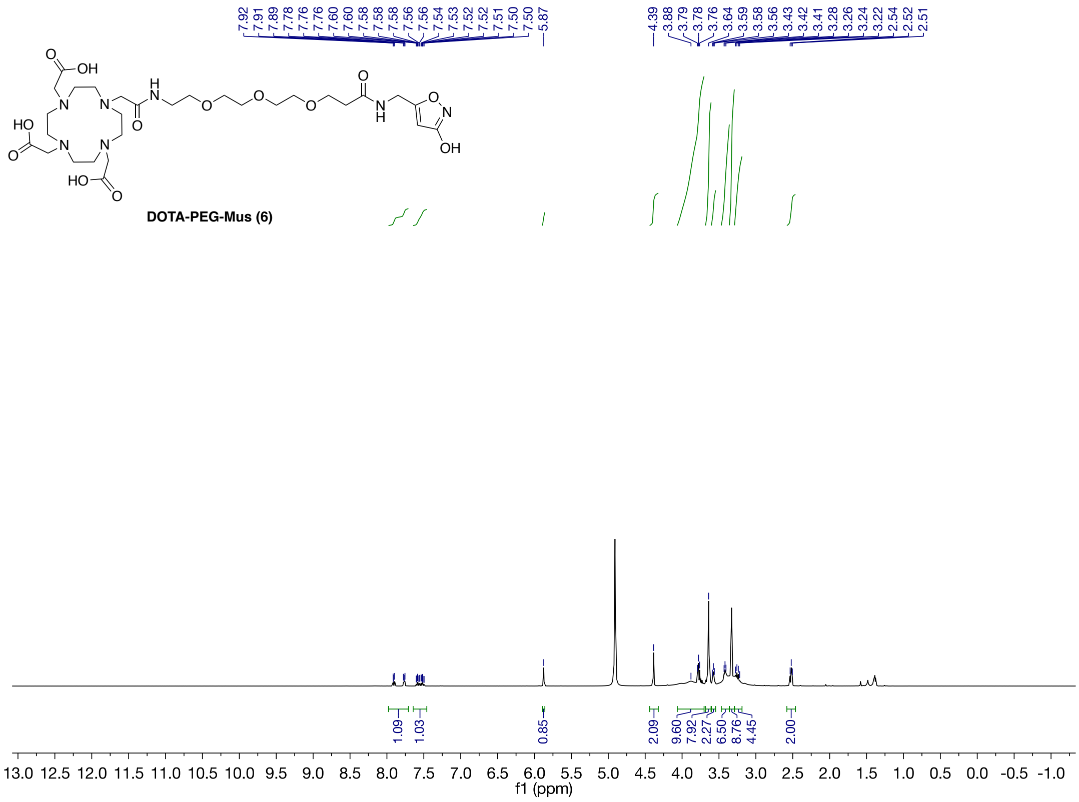

^{13}C NMR spectrum of DOTA-PEG-Mus

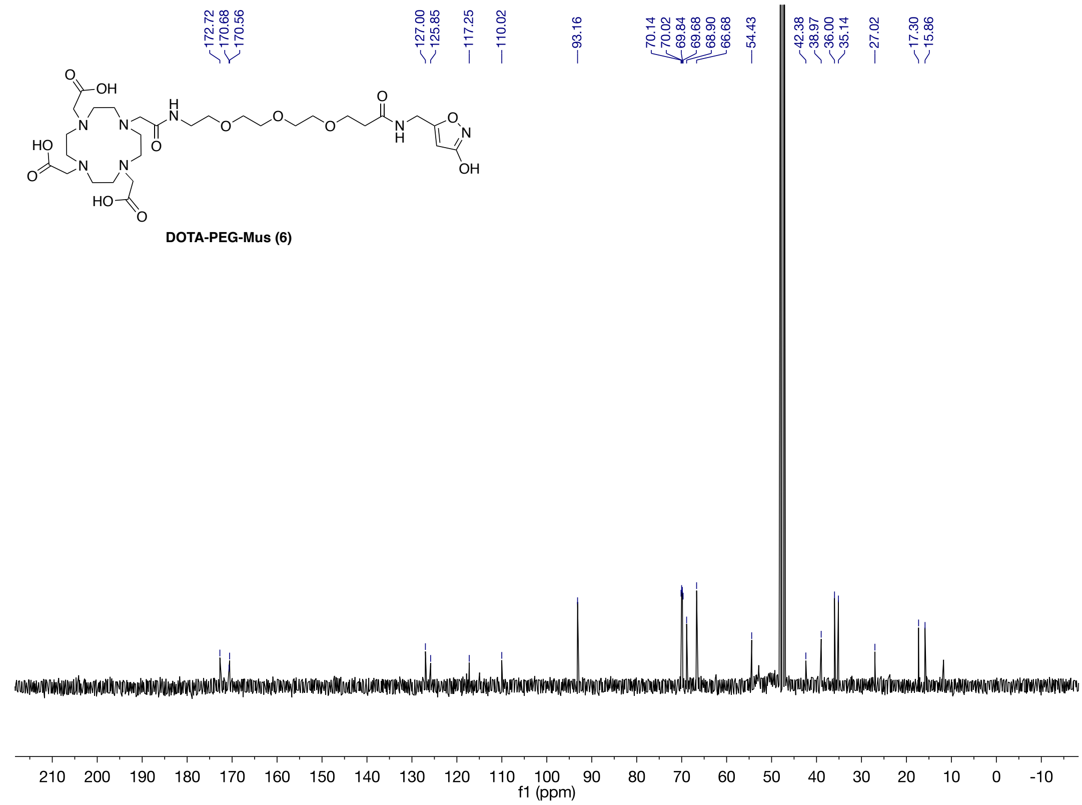

Supplement: Supplementary file 1 — Supplementary Information [file 41467_2019_13933_MOESM1_ESM.pdf]
