# Supplementary material for: Image-guided neural activity manipulation with a paramagnetic drug
Source: Nat Commun. 2020 Jan 9;11:136. doi: 10.1038/s41467-019-13933-5 (PMC6952355; doi:10.1038/s41467-019-13933-5)
Supplement: Supplementary file 2 — Reporting Summary [file 41467_2019_13933_MOESM2_ESM.pdf]

nature research

Corresponding author(s): ALAN JASANOFF

Last updated by author(s): Nov 22, 2019

Reporting Summary

Nature Research wishes to improve the reproducibility of the work that we publish. This form provides structure for consistency and transparency in reporting. For further information on Nature Research policies, see Authors & Referees and the Editorial Policy Checklist.

Statistics

For all statistical analyses, confirm that the following items are present in the figure legend, table legend, main text, or Methods section.

n/a	Confirmed	
☐	☒	The exact sample size (n) for each experimental group/condition, given as a discrete number and unit of measurement
☐	☒	A statement on whether measurements were taken from distinct samples or whether the same sample was measured repeatedly
☐	☒	The statistical test(s) used AND whether they are one- or two-sided<br>*Only common tests should be described solely by name; describe more complex techniques in the Methods section.*
☒	☐	A description of all covariates tested
☒	☐	A description of any assumptions or corrections, such as tests of normality and adjustment for multiple comparisons
☐	☒	A full description of the statistical parameters including central tendency (e.g. means) or other basic estimates (e.g. regression coefficient) AND variation (e.g. standard deviation) or associated estimates of uncertainty (e.g. confidence intervals)
☐	☒	For null hypothesis testing, the test statistic (e.g. F, t, r) with confidence intervals, effect sizes, degrees of freedom and P value noted<br>*Give P values as exact values whenever suitable.*
☒	☐	For Bayesian analysis, information on the choice of priors and Markov chain Monte Carlo settings
☒	☐	For hierarchical and complex designs, identification of the appropriate level for tests and full reporting of outcomes
☒	☐	Estimates of effect sizes (e.g. Cohen's d, Pearson's r), indicating how they were calculated

Our web collection on statistics for biologists contains articles on many of the points above.

Software and code

Policy information about availability of computer code

Data collection	Paravision
Data analysis	Matlab, AFNI

For manuscripts utilizing custom algorithms or software that are central to the research but not yet described in published literature, software must be made available to editors/reviewers. We strongly encourage code deposition in a community repository (e.g. GitHub). See the Nature Research guidelines for submitting code & software for further information.

Data

Policy information about availability of data

All manuscripts must include a data availability statement. This statement should provide the following information, where applicable:

- Accession codes, unique identifiers, or web links for publicly available datasets
- A list of figures that have associated raw data
- A description of any restrictions on data availability

Raw MRI datasets generated during and/or analyzed during the current study are available from the corresponding author on reasonable request.

Field-specific reporting

Please select the one below that is the best fit for your research. If you are not sure, read the appropriate sections before making your selection.

☒ Life sciences ☐ Behavioural & social sciences ☐ Ecological, evolutionary & environmental sciences

For a reference copy of the document with all sections, see nature.com/documents/nr-reporting-summary-flat.pdf

Life sciences study design

All studies must disclose on these points even when the disclosure is negative.

Sample size	Replicates were performed to ensure reproducibility of measurements, not to meet explicit statistical criteria.
Data exclusions	N/A
Replication	As described in text.
Randomization	N/A
Blinding	N/A

Reporting for specific materials, systems and methods

We require information from authors about some types of materials, experimental systems and methods used in many studies. Here, indicate whether each material, system or method listed is relevant to your study. If you are not sure if a list item applies to your research, read the appropriate section before selecting a response.

Materials & experimental systems

n/a	Involved in the study
☒	☐ Antibodies
☒	☐ Eukaryotic cell lines
☒	☐ Palaeontology
☐	☒ Animals and other organisms
☒	☐ Human research participants
☒	☐ Clinical data

Methods

n/a	Involved in the study
☒	☐ ChIP-seq
☒	☐ Flow cytometry
☐	☒ MRI-based neuroimaging

Animals and other organisms

Policy information about studies involving animals; ARRIVE guidelines recommended for reporting animal research

Laboratory animals	Nine male Sprague-Dawley rats (300-400 g) were used for MRI studies. Additional animals supplied primary cortical neurons for electrophysiology tests in vitro.
Wild animals	N/A
Field-collected samples	N/A
Ethics oversight	All animal procedures were performed in strict compliance with US Federal guidelines, with oversight by the MIT Committee on Animal Care

Note that full information on the approval of the study protocol must also be provided in the manuscript.

Magnetic resonance imaging

Experimental design

Design type	Block design
Design specifications	10 s forepaw stimulation, alternating with 40 s rest
Behavioral performance measures	N/A

Acquisition

Imaging type(s)	Anatomical and functional
Field strength	9.4 T
Sequence & imaging parameters	A rapid acquisition with refocused echoes (RARE) pulse sequence were used to acquire T2-weighted anatomical images, with number of averages (NA) = 6, matrix size = 300 × 200, FOV = 3 cm × 2 cm, slice thickness = 1.2 mm, TR = 5000 ms, effective TE = 60 ms, and RARE factor = 8. To quantify the extent of ParaMus infusion, T1-weighted RARE images were

also acquired, using NA = 6, matrix size = 300 × 200, FOV = 3 cm × 2 cm, slice thickness = 1.2 mm, TR = 5000 ms, effective TE = 60 ms, and RARE factor = 8. For functional imaging, echo planar imaging (EPI) image series were acquired during alternating blocks of forepaw stimulation and rest. EPI scan parameters were NA = 6, matrix size = 300 × 200, FOV = 3 cm × 2 cm, slice thickness = 1.2 mm, TR = 5000 ms, effective TE = 60 ms, RARE factor = 8.

Area of acquisition: Whole brain

Diffusion MRI: ☐ Used ☒ Not used

Preprocessing

Preprocessing software: Paravision, AFNI

Normalization: Spatial registration was performed. Effects were normalized to baseline and in some cases pre-treatment values

Normalization template: Rat atlas

Noise and artifact removal: The time series data from the EPI scans were smoothed with a Gaussian spatial kernel of 1 mm full width at half-maximum prior to statistical analysis, and each voxel time course was subsequently temporally smoothed using a sliding box window of width 3.

Volume censoring: Outlier scans detected by median absolute deviation from time series trends in each data set were censored from the analysis.

Statistical modeling & inference

Model type and settings: General linear model with fixed effects.

Effect(s) tested: Stimulus responses, with motion correlates regressed out

Specify type of analysis: ☐ Whole brain ☐ ROI-based ☒ Both

Anatomical location(s): ROI analysis focused on the somatosensory cortex forelimb region (S1FL)

Statistic type for inference (See Eklund et al. 2016): F-test

Correction: Partial Bonferroni

Models & analysis

n/a	Involved in the study
☒	☐ Functional and/or effective connectivity
☒	☐ Graph analysis
☒	☐ Multivariate modeling or predictive analysis
